# Epitope analysis of an anti-mouse CCR1 monoclonal antibody S15040E using flow cytometry

**DOI:** 10.1016/j.bbrep.2025.102265

**Published:** 2025-09-13

**Authors:** Ayaka Okada, Hiroyuki Suzuki, Takao Arimori, Tomohiro Tanaka, Mika K. Kaneko, Yukinari Kato

**Affiliations:** a Department of Antibody Drug Development, Tohoku University Graduate School of Medicine, 2-1 Seiryo-machi, Aoba-ku, Sendai, Miyagi, 980-8575, Japan; b Institute for Protein Research, Osaka University, 3-2. Yamadaoka, Suita, Osaka, 565-0871, Japan

**Keywords:** Mouse CCR1, Monoclonal antibody, Epitope mapping, Alanine scanning, Flow cytometry

## Abstract

The C–C motif chemokine receptor 1 (CCR1) is widely expressed in various immune cells and plays crucial roles in the maturation and migration of immune cells. CCR1 has been considered an attractive drug target for treating autoimmune diseases and tumors. An anti-mouse CCR1 (mCCR1) monoclonal antibody (clone S15040E) has been used in various *in vivo* studies to identify mCCR1-positive cells by flow cytometry. However, the binding epitope has not been determined. This study investigated the binding epitope of S15040E using flow cytometry. The mCCR1 extracellular domain-substituted mutant analysis showed that S15040E recognizes the extracellular loop 2 (ECL2, aa 172–197) of mCCR1. Next, alanine (or glycine) scanning was conducted in the ECL2 region. The results revealed that Trp176, Phe178, and Arg181 are essential amino acids for the recognition by S15040E. These results showed the involvement of the ECL2 of mCCR1 in the recognition by S15040E.

## Highlights

• An anti-mouse CCR1 monoclonal antibody (S15040E) has been used in flow cytometry.
• S15040E recognizes a conformational epitope on the extracellular loop 2 (ECL2, aa 172–197) of mouse CCR1.
• The Trp176, Phe178, Arg181, and Cys183 in ECL2 are essential for the recognition by S15040E.
• S15040E may contribute to the structural analysis of mouse CCR1 to understand the function.

## 1 Introduction

Chemokine receptors belong to class A seven transmembrane (7TM) receptors and play an essential role in guiding leukocyte trafficking in immune surveillance and inflammatory response [1]. The cognate chemokines are named according to the sequence of the first two cysteines (CC, CXC, XC, or CX3C motif). The C–C motif chemokine ligands (CCL1 to CCL28) are recognized by C–C chemokine receptor type 1 (CCR1) to CCR10 [2]. Upon ligand binding, chemokine receptors typically activate G protein pathways and recruit β-arrestins [3,4].

CCR1 mediates inflammatory responses and plays an essential role in the development of autoimmune diseases [1,5]. It has been considered an attractive drug target for treating allergic and autoimmune diseases [6]. Among the chemokine receptors, CCR1 possesses ligand promiscuity, which allows it to recognize at least nine human CC chemokines, including CCL3, CCL5, CCL7, CCL8, CCL13–16, and CCL23 [2,7,8].

The structural understanding of the chemokine receptor activation is essential for the development of therapeutic agents in the chemokine system. Among the CCR family members, CCR2 and CCR5 have been characterized in both inactive and the ligand-bound active states [[9], [10], [11], [12]], while ligand-bound active-state of CCR8 and CCR6 and inactive-state of CCR7 and CCR9 structures were also determined [[13], [14], [15], [16]]. Furthermore, the CCL15-CCR1 complex showed crucial sequences in extracellular loop (ECL) 2–3 for ligand binding distinct from many other chemokine–receptor complexes, which provided new insights into the mode of chemokine recognition [13]. Moreover, the structures of CCR8 in complex with either the endogenous ligand CCL1 or the antagonistic monoclonal antibody (mAb) were solved, which provides the specific activation mechanism by CCL1 and inhibition by mAb [17]. Therefore, anti-chemokine receptor mAbs with defined epitopes are helpful for the analysis of particular structures.

An anti-mouse CCR1 (mCCR1) mAb (clone S15040E) has been used in various *in vivo* studies to identify mCCR1-positive cells using flow cytometry [[18], [19], [20]]. However, the binding epitope has not been determined. MAbs against 7TM proteins sometimes recognize conformational epitopes but not linear epitopes [17]. In this case, we have faced difficulty determining the epitopes using conventional methods such as enzyme-linked immunosorbent assays. This study investigated the binding epitope of S15040E using flow cytometry-based approaches.

## 2 Materials and methods

### 2.1 Plasmid construction

pCAG-Ble-mCCR1 and pCAG-Ble-mouse CCR5 (mCCR5) were generated as previously described [21,22]. Chimeric mutants including mCCR5 (mCCR1p2–34), mCCR5 (mCCR1p92–107), mCCR5 (mCCR1p172–197), and mCCR5 (mCCR1p265–281) with an N-terminal PA16 tag were produced as described previously [23]. Alanine (or glycine)-substituted mutants of mCCR1 with or without an N-terminal PA16 tag were constructed using QuikChange Lightning Site-Directed Mutagenesis Kits (Agilent Technologies Inc., Santa Clara, CA, USA). PCR fragments bearing the desired mutations were inserted into the pCAG-Ble or pCAG-neo vectors (FUJIFILM Wako Pure Chemical Corporation, Osaka, Japan).

### 2.2 Cell lines

The chimeric and the point mutant plasmids were transfected into Chinese hamster ovary (CHO)–K1 cells (American Type Culture Collection, Manassas, VA, USA) using the Neon Transfection System (Thermo Fisher Scientific Inc., Waltham, MA, USA).

### 2.3 Antibodies

An anti-mCCR1 mAb (clone S15040E) was purchased from BioLegend (San Diego, CA, USA). C_1_Mab-6 was established as described previously [21]. NZ-1 (an anti-PA16 tag mAb) was described previously [24].

### 2.4 Flow cytometry

Cells were harvested after brief exposure to 0.25 % trypsin/1 mM ethylenediaminetetraacetic acid (Nacalai Tesque, Inc.). After washing with 0.1 % bovine serum albumin in phosphate-buffered saline, cells (2 × 10^5^) were treated with S15040E (1 μg/mL) or NZ-1 (1 μg/mL) for 30 min at 4 °C and subsequently with Alexa Fluor 488-conjugated anti-rat IgG (1:2000; Cell Signaling Technology, Inc., Danvers, MA, USA). Fluorescence data (total 10,000 cells/samples) were obtained using the SA3800 Cell Analyzer (Sony Corp., Tokyo, Japan). Using FlowJo software (BD Biosciences, Franklin Lakes, NJ, USA), single cells were selected by gating: side scatter versus forward scatter. The fluorescence intensity was plotted, and the geometric mean was determined.

### 2.5 Structural prediction of mCCR1 using AlphaFold3

The three-dimensional structures of wild-type mCCR1 and its alanine (or glycine)-substituted mutants were predicted using the online tool AlphaFold3 (https://alphafoldserver.com/) [25].

## 3 Results

### 3.1 Determination of the epitope of an anti-mCCR1 mAb, S15040E, by flow cytometry using chimeric proteins

**Fig. 1:**
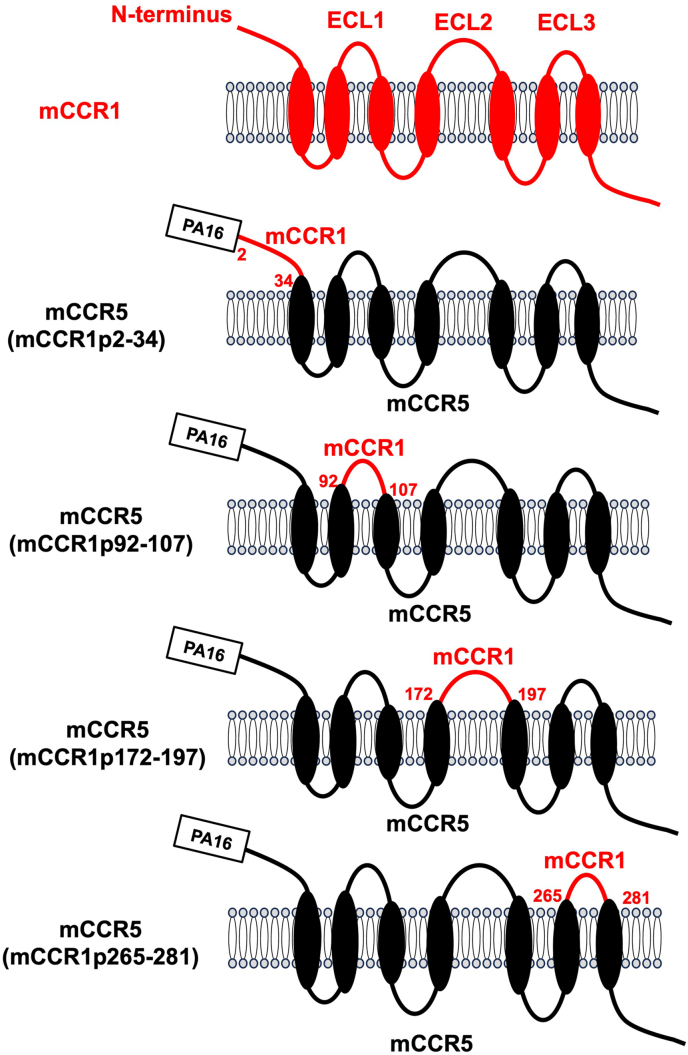
**Schematic illustration of chimeric proteins.** The four extracellular regions of mCCR1, including the N-terminal region (residues 2–34), ECL1 (residues 92–107), ECL2 (residues 172–197), and ECL3 (residues 265–281) were substituted into the corresponding regions of mCCR5. ECL, extracellular loop.

**Fig. 2:**
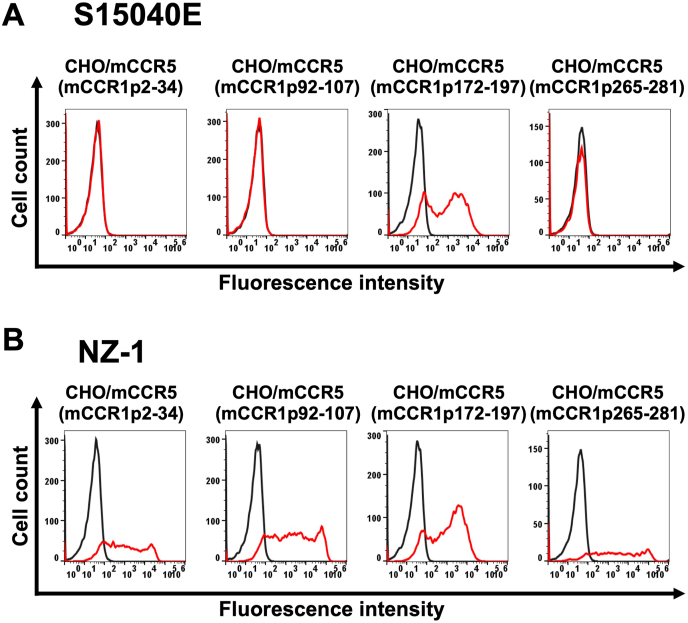
**Determination of the epitope of an anti-mCCR1 mAb, S15040E, by flow cytometry using chimeric proteins.** CHO–K1 transiently expressed the chimeric proteins were treated with S15040E (1 μg/mL, A), an anti-PA16 tag mAb, NZ-1 (1 μg/mL, B), or blocking buffer for 30 min at 4 °C, followed by the addition of Alexa Fluor 488-conjugated anti-rat IgG. Red lines show the cells with S15040E or NZ-1 treatment, and black lines show cells treated with a blocking buffer as a negative control.

**Supplementary Fig. S1:**
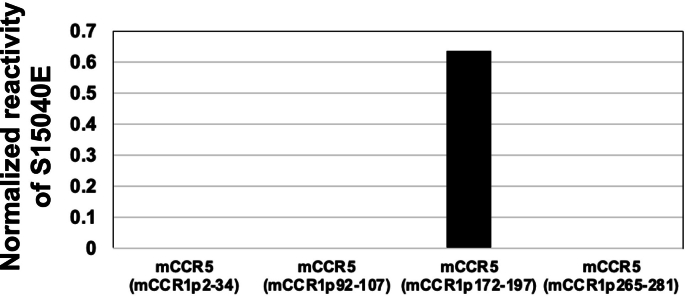
**The normalized reactivity of S15040E in**Fig. 2**.** The values (geometric mean of S15040E / geometric mean of NZ-1) were determined in chimeric proteins.

An anti-mCCR1 mAb (clone S15040E) is applicable for flow cytometry. To investigate the binding epitope of S15040E, we focused on four extracellular regions of mCCR1, including the N-terminal region (aa 2–34), ECL1 (aa 92–107), ECL2 (aa 172–197), and ECL3 (aa 265–281). The four extracellular regions of mCCR1 were substituted into the corresponding regions of mCCR5, which possesses a high sequence homology to mCCR1. As shown in Fig. 1, plasmids encoding mCCR5 (mCCR1p2–34), mCCR5 (mCCR1p92–107), mCCR5 (mCCR1p172–197), and mCCR5 (mCCR1p265–281) were generated. The chimeric proteins were transiently expressed on CHO–K1 cells, and the reactivities to S15040E were analyzed using flow cytometry (Fig. 2A). S15040E reacted with mCCR5 (mCCR1p172–197), but not with mCCR5 (mCCR1p2–34), mCCR5 (mCCR1p92–107), and mCCR5 (mCCR1p265–281) (Fig. 2A). The cell surface expression of each mutant was confirmed by an anti-PA16 tag mAb, NZ-1 (Fig. 2B). The normalized reactivity of S15040E was shown in Supplementary Fig. S1. These results indicated that S15040E recognizes the ECL2 of mCCR1.

### 3.2 Determination of the S15040E epitope by flow cytometry using alanine scanning

**Fig. 3:**
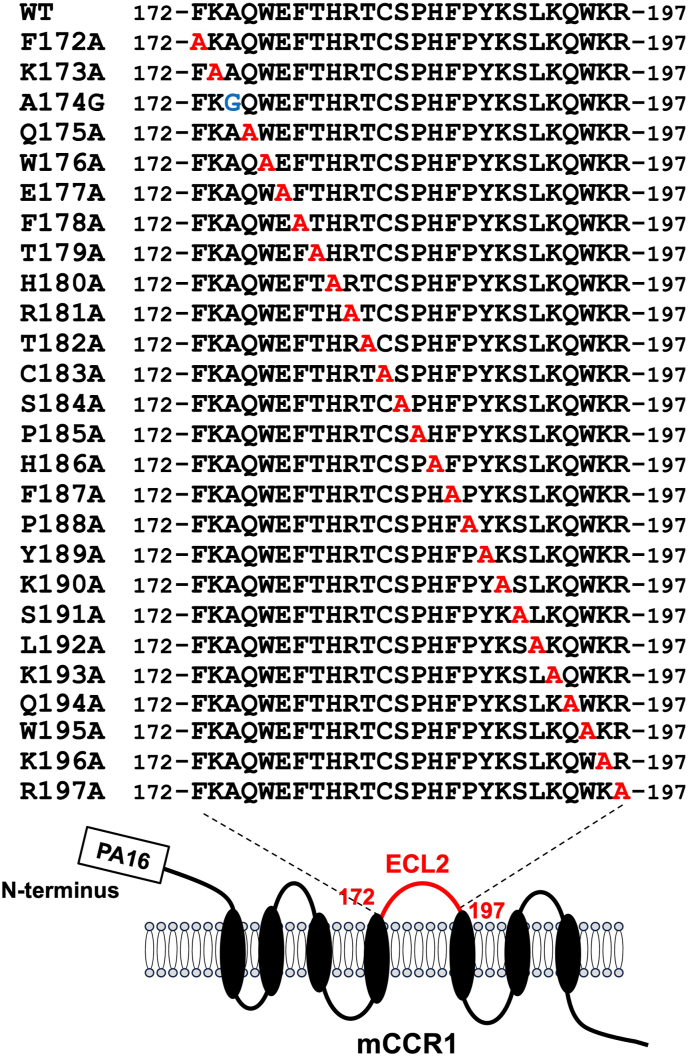
The illustration of alanine (or glycine) substituted mutants of mCCR1.

**Fig. 4:**
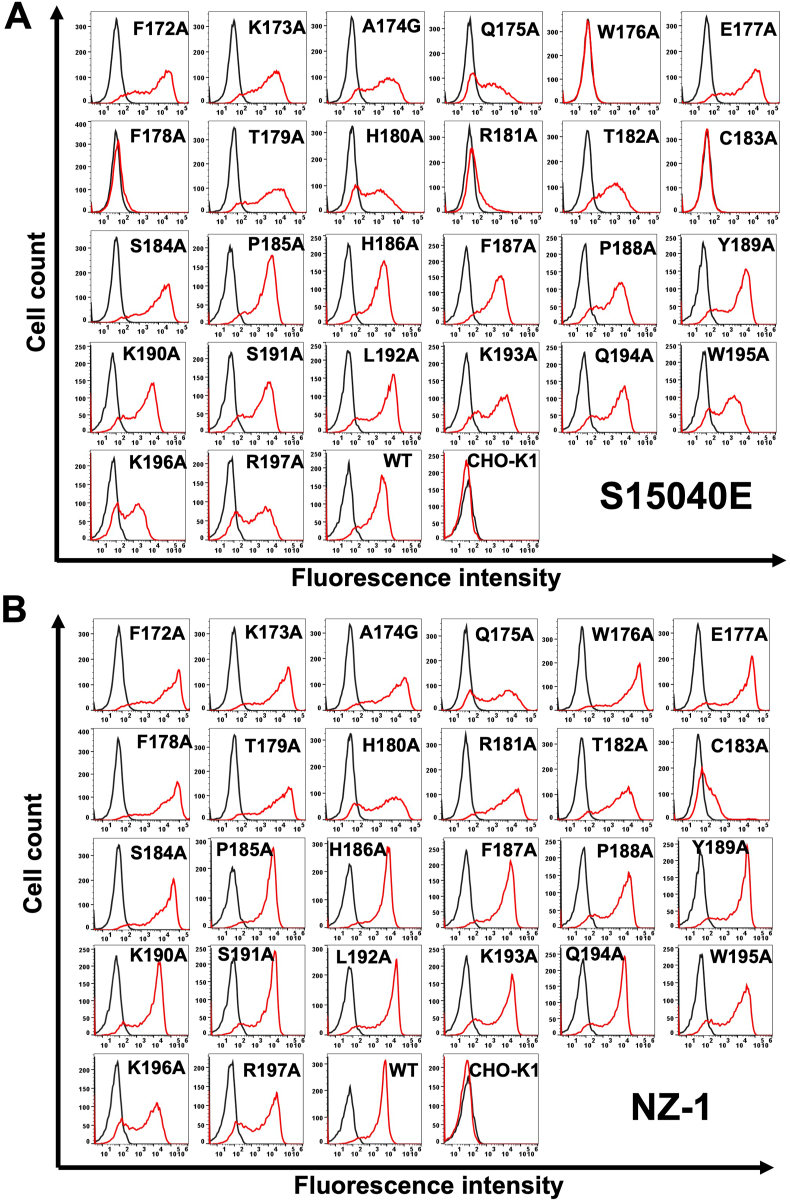
**Determination of the S15040E epitope by flow cytometry using alanine scanning.** CHO–K1 transiently expressed PA16-tagged mCCR1 mutants and wild-type (WT) were treated with S15040E (1 μg/mL, A), NZ-1 (1 μg/mL, B), or blocking buffer for 30 min at 4 °C. Then, cells were treated with Alexa Fluor 488-conjugated anti-rat IgG. Red lines show the cells with S15040E or NZ-1 treatment, and black lines show cells treated with a blocking buffer as a negative control.

**Fig. 5:**
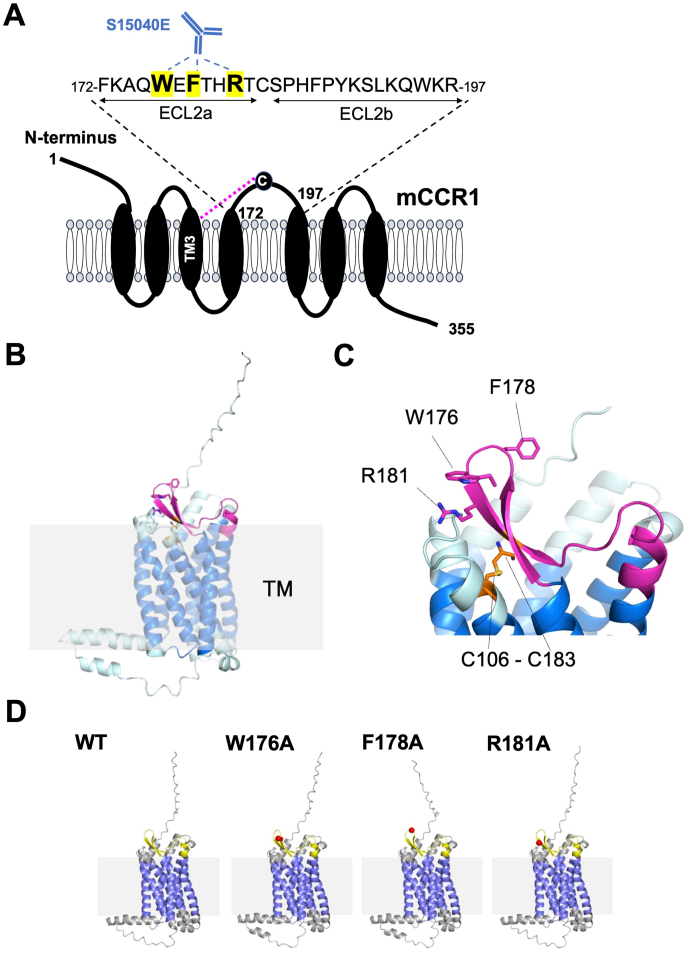
**The schematic illustration of the S15040E epitope.** (A) Trp176, Phe178, and Arg181 are essential amino acids for the recognition by S15040E. The dotted magenta line represents the disulfide bridge between TM3 and ECL2. TM3, transmembrane helix 3; ECL, extracellular loop. (B) Prediction of the three-dimensional structure of WT mCCR1 using AlphaFold3. (C) The epitope amino acids and a predicted disulfide bond are shown. The transmembrane region and the ECL2 region are shown in blue and magenta, respectively. (D) Comparison of the three-dimensional structure of WT mCCR1 with the three mutants (W176A, F178A, and R181A) using AlphaFold3. The transmembrane region and the ECL2 are shown in blue and yellow, respectively. Mutated sites are indicated by red spheres.

**Supplementary Fig. S2:**
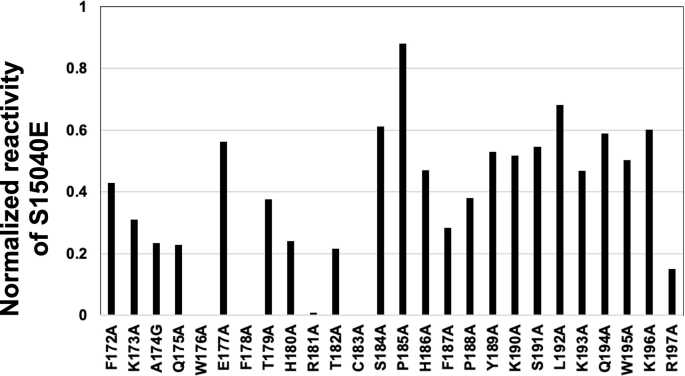
**The normalized reactivity of S15040E in**Fig. 4**.** The values (geometric mean of S15040E / geometric mean of NZ-1) were determined in mCCR1 point mutants.

**Supplementary Fig. S3:**
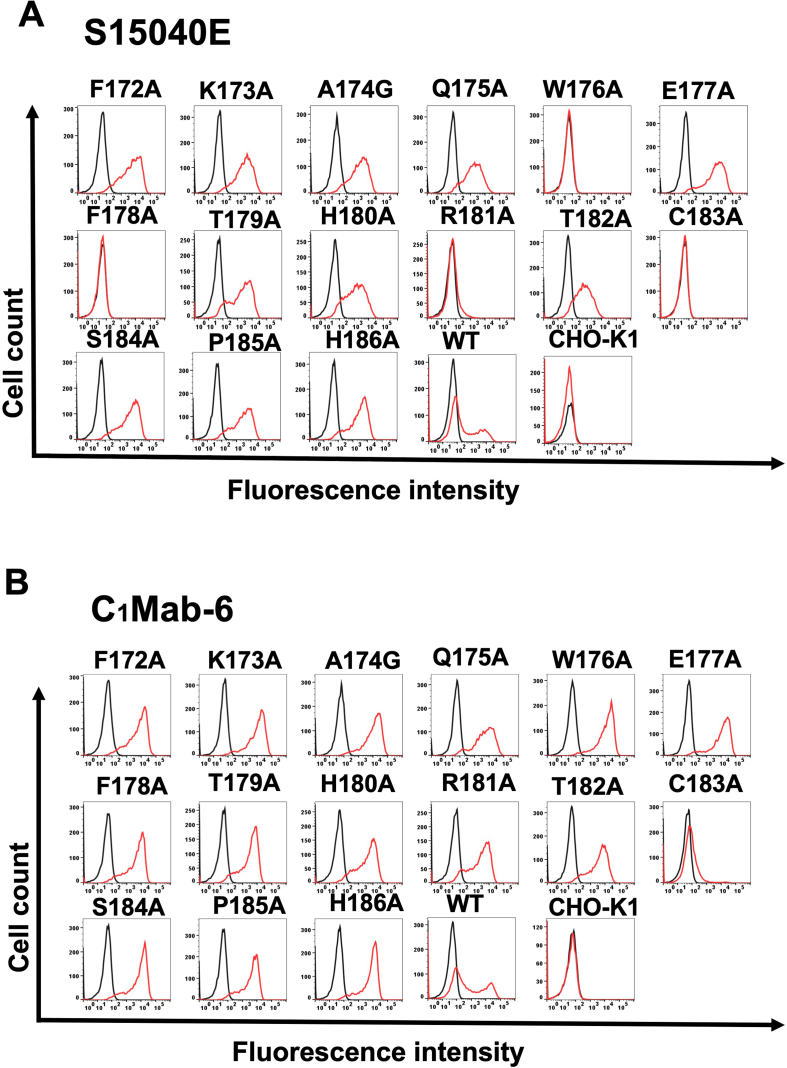
**The reactivity of another anti-mCCR1 mAb, C_1_Mab-6 to mCCR1 mutants.** CHO–K1 transiently expressed mCCR1 mutants and wild-type (WT) were treated with S15040E (1 μg/mL, A), C_1_Mab-6 (1 μg/mL, B), or blocking buffer for 30 min at 4 °C. Then, cells were treated with Alexa Fluor 488-conjugated anti-rat IgG. Red lines show the cells with C_1_Mab-6 or S15040E treatment, and black lines show cells treated with a blocking buffer as a negative control.

Next, alanine scanning was conducted in the ECL2 of mCCR1. Twenty-six alanine (or glycine) substituted mutants of mCCR1 were constructed (Fig. 3), and the mutant proteins were transiently expressed in CHO–K1 cells. The reactivity against S15040E was assessed using flow cytometry. As shown in Fig. 4A, S15040E did not react with four mutants (W176A, F178A, R181A, and C183A). In contrast, S15040E reacted with the other twenty-two mutants. The cell surface expression of each mutant was confirmed by NZ-1. However, the expression of C183A was low compared to others (Fig. 4B). The normalized reactivity of S15040E was shown in Supplementary Fig. S2. Similar results were obtained by mCCR1 without PA16 tag (Supplementary Fig. S3A). We found that the three mutants (Trp176, Phe178, and Arg181) were recognized by another anti-mCCR1 mAb, C_1_Mab-6 [21]. However, the reactivity to C183A was quite low compared to others (Supplementary Fig. S3B). These result suggested that three point mutations (Trp176, Phe178, and Arg181) did not affect the overall structure of mCCR1. In contrast, C183A should not be included as an epitope amino acid because we cannot exclude the possibility that the overall structure of mCCR1 was disrupted by C183A mutation. Fig. 5A summarized the critical amino acids for S15040E binding. Furthermore, the three-dimensional structures of wild-type (WT) mCCR1 (Fig. 5B) and the epitope of S15040E (Fig. 5C) were shown using AlphaFold3. We compared the structure of WT mCCR1 with its alanine (or glycine)-substituted mutants. As shown in Fig. 5D, the three mutants (W176A, F178A, and R181A) were predicted to maintain the overall structure.

## 4 Discussion

CCR1 has been considered a drug target for treating allergic and autoimmune diseases [6]. Recently, blockade of CCR1 by a neutralizing anti-CCR1 mAb in myeloid cells showed the therapeutic efficacy in a mouse model of colorectal cancer [26]. This study demonstrated the flow cytometry-based epitope mapping of an anti-mouse CCR1 mAb (S15040E) using the chimeric proteins (Fig. 1, Fig. 2). Furthermore, we determined that the Trp176, Phe178, and Arg181 in ECL2 are essential for the recognition by S15040E in alanine scanning (Fig. 3, Fig. 4). We previously determined the epitope of an anti-mouse CCR8 mAb, C_8_Mab-2 [23]. Our strategy for epitope identification would contribute to the understanding of mAb-epitope interaction.

The 7TM receptors have a conserved disulfide bridge between transmembrane helix 3 (TM3) and ECL2 [27]. The Cys183 is well conserved, and the sole cysteine in ECL2 forms a disulfide bridge with TM3 (Fig. 5A and C). Since the mCCR1 C183A mutant was not recognized some mAbs including NZ-1 and C_1_Mab-6, the structure might be disrupted by the loss of disulfide bridge.

ECL2 is essential for interaction with chemokines and is the largest region covering the activation-associated receptor binding pocket. Several anti-chemokine receptor mAbs recognize the ECL2 [17]. ECL2 is divided into two parts before and after the disulfide bridge (ECL2a and ECL2b, respectively). Both parts involve the chemokine signaling selectivity and pharmacological activity [[28], [29], [30], [31]]. The Trp176, Phe178, and Arg181 are in the ECL2a of mCCR1. In human CCR1, the ECL2a is essential for the recognition of CCL15 [13]. Therefore, S15040E may possess neutralization activity against the ECL2a-bound ligands.

G protein-coupled receptors can transduce intracellular signaling through G proteins and β-arrestins. "Balanced" agonists or antagonists can activate or inhibit these signaling pathways. In contrast, specific pathways can be selectively triggered in a "biased" response. The biased responses can arise from biased ligands or biased receptors, all of which can drive preferential activation of either G protein- or β-arrestin-mediated pathways [32]. CCR1 is known to be a biased receptor that can selectively activate G proteins or β-arrestin pathways by diverse CCL15 isoforms [13,33]. Further structural analysis of the S15040E-mCCR1 complex may provide new insights into the mechanism of biased response and the development of therapeutic drugs.

## CRediT authorship contribution statement

**Ayaka Okada:** Investigation. **Hiroyuki Suzuki:** Writing – original draft. **Takao Arimori:** Formal analysis, Visualization. **Tomohiro Tanaka:** Investigation, Funding acquisition. **Mika K. Kaneko:** Conceptualization. **Yukinari Kato:** Writing – review & editing, Project administration, Funding acquisition, Conceptualization.

## Author disclosure statement

The authors have no conflict of interest.

## Funding information

This research was supported in part by Japan Agency for Medical Research and Development (AMED) under Grant Numbers: JP25am0521010 (to Y.K.), JP25ama121008 (to Y.K.), JP25ama221339 (to Y.K.), and JP25bm1123027 (to Y.K.), and by the Japan Society for the Promotion of Science (JSPS) Grants-in-Aid for Scientific Research (KAKENHI) grant nos. 24K18268 (to T.T.) and 25K10553 (to Y.K.).

## Declaration of competing interest

The authors declare that they have no known competing financial interests or personal relationships that could have appeared to influence the work reported in this paper.

## Data Availability

Data will be made available on request.
